# Social and clinical attributes of patients who restart antiretroviral therapy in central and Copperbelt provinces, Zambia: a retrospective longitudinal study

**DOI:** 10.1186/s12889-016-2922-3

**Published:** 2016-03-29

**Authors:** Chama Mulubwa, Oliver Mweemba, Patrick Katayamoyo, Hikabasa Halwindi

**Affiliations:** Department of Public Health, School of Medicine, University of Zambia, PO Box 50110, Lusaka, Zambia; FHI 360, Plot 2374, Farmers Village, ZNFU Complex, Lusaka, Zambia

**Keywords:** CD4 cell count, Antiretroviral therapy, Restart, Defaulting, Lost to follow-up, Zambia

## Abstract

**Background:**

About 30 % of the patients initiated on antiretroviral therapy in Zambia default treatment. Some of these patients later restart treatment; however, the characteristics of these patients have not been well described and documented. The aim of this study was to describe and document the socio-demographic and clinical characteristics of patients who default and restart antiretroviral therapy, and to determine the socio-demographic characteristics associated with CD4 count response at 6 and 24 months of restarting antiretroviral therapy.

**Methods:**

A longitudinal retrospective analysis was performed on data from 535 adult patients restarting antiretroviral therapy in 2009 and 2010 at five antiretroviral therapy centres in Copperbelt and Central provinces of Zambia. To determine the association between the socio-demographic characteristics and CD4 cell count, quantile regression models were used.

**Results:**

Older age above 45 years was associated with a significantly lower CD4 cell response by 38.1 cells/mm^3^ (95 % Confidence interval [CI]: −109.4 to −0.2) compared to the younger age (15–29 years). Patients in formal employment (Adjusted Coefficient [AC] 29.5, 95 % CI: 22.8 to 81.1) and self-employment (AC 48.1, 95 % CI: 18.6 to 77.4) gained significantly higher CD4 cells than those unemployed. In addition, baseline CD4 count, type of treatment, WHO staging, total duration on treatment and duration lost to follow-up were found to be strong predictors of CD4 cell count at 6 and 24 months after restarting antiretroviral therapy treatment.

**Conclusion:**

Age and occupation were the only socio-demographic characteristics predicting CD4 count in the patients at 6 months after restarting antiretroviral therapy after adjusting for other confounding clinical variables.

## Background

Antiretroviral therapy (ART) is an essential part of the fight against the human immunodeficiency virus (HIV) and acquired immunodeficiency syndrome (AIDS), sustains human lives for people living with HIV (PLHIV) and prevents HIV/AIDS morbidity and mortality [1]. ART though expensive, in the long run saves money and promotes development [2]. While the benefits and successes of ART have been widely acknowledged worldwide [1, 3–6], about 30 % of the patients are lost to follow-up (LTFU) from treatment in programmes in most developing countries [7–10]. However, some of the patients who interrupt ART later restart the treatment. This interruption of ART prevents patients from reaping the full benefits of the treatment [2].

LTFU was defined in the 2010 Zambia ART guidelines as missing a scheduled pharmacy pick-up for more than 60 days [11]. Patients who came back to ART after LTFU were considered to have restarted ART. Therefore, treatment resumption in this study was defined as restarting ART after the patient was declared LTFU at the health facility. Defaulting results in treatment interruption, which has negative effects on the CD4 cell response of the patient. Patients who default and restart ART run a higher risk of illness and death because of AIDS-related conditions [3, 4, 10, 12–18]. Defaulting may, therefore, result in patients restarting ART at an increased viral rebound, acute retroviral syndrome due to immune decomposition and an increased risk of HIV transmission [19].

It has been suggested that patients who restart ART normally resume treatment at a CD4 count level lower to their CD4 count prior to ART defaulting but similar to the CD4 count at first ART initiation [17]. This underscores the potentially negative impact of treatment defaulting that might lead to a reversal of the immunological recovery made while on the initial treatment [20]. Defaulting ART has also been associated with age, gender, residency and self-rate health. In some studies Younger age or female gender or patients with CD4 counts >350 cells/mm^3^ were found to be more likely to default. Other studies established positive significant associations between restarting ART with older age or female gender or urban residency or shorter time since defaulting [18, 21–23]. Despite knowing the factors associated with defaulting and restarting ART, the social and clinical characteristics of such patients who default and restart have not been well described and documented in Zambia where the attrition rate of patients is still high [8]. Therefore this study aimed at describing and documenting the socio-demographic and clinical characteristics of patients who defaulted and restarted ART from January, 2009 to December, 2010 in Zambia; and also to determine the socio-demographic and clinical characteristics associated with CD4 count response at 6 and 24 months after restarting ART.

## Methods

### Study design and sampling

A retrospective longitudinal study design was used. Patients who restarted ART between January 2009 and December 2010 were sampled from five Ministry of Health-Owned ART health facilities that were supported by the FHI 360-led Zambia Prevention, Care and Treatment project. These ART facilities were purposively selected from the Central and Copperbelt provinces because of the high restarting rates recorded during the selected period. The health facilities selected in Central Province were Liteta Hospital, Kabwe General Hospital and Mwachisompola Demo Zone and in Copperbelt Province Kitwe Central Hospital and Chipokota Mayamba Health Centre. At these five selected ART facilities, all adult patients, male and female ≥16 years who were restarted on ART in the period January 2009 to December 2010 were included in the study. Paediatric patients (below 15 years), pregnant women and patients transferred in and out during the study period were excluded.

### Study population

Women and men enrolled in the ART care and reported to have defaulted and restarted ART services from January 2009 to December 2010 were selected for this study. All the five ART facility chosen were high volume facilities and served geographically and socially discreet catchment populations and received referrals from smaller health facilities within their catchment areas. ART health care services were accessible and ran throughout the week as part of the primary health services provided by the hospitals. The population was selected because they had access to a comprehensive package of ART care, which included HIV counselling and testing and provision of ART.

### Data collection

A data extraction sheet was used to excerpt the patient data from the FHI 360 supported database (Smartcare) at the ART facilities. Smartcare is an electronic database system where all the records collected from the patients at the ART clinic were entered and stored. For clinic reviews five Smartcare forms were used and these were: the Patient Locator form used to take the patient details about residency and next of kin; the Initial History and Patient form where the initial detailed history of the patient was recorded before they were initiated on ART; Eligibility/Initiation form which was used to asses eligibility for ART; the Clinical Follow-up form used for subsequent reviews once the patient was initiated on ART and the Patient Status which was filed in when the status (such as defaulting, restarting, transfer and or death) of the patient changed.

The extraction of data was done by trained FHI 360 data collectors, stationed at the ART facilities selected in this study. The data collection process included the review of the laboratory forms in the patient file and five Smartcare forms which were either electronic or paper based.

All the patients listed in Smartcare, who had defaulted and restarted ART were eligible for the study. A complete list of 535 patients who were restarted on ART during the period of interest was generated by running a Data Quality Report (DQR) in Smartcare which gave a list of names and patient file numbers of all those who were recorded as restarted after having defaulted ART. The file numbers from the generated list were then used to consolidate both the electronic and paper based patient files. After merging both the Smartcare and paper-bases patient files, the information required was then collected using the data collection form. This data for each patient was retrospectively collected at 6 months and 24 months after restarting ART.

### Ethical issues

The ERES Coverage ethics committee approved this study. Permission to conduct the study was granted by FHI 360 Zambia, Ministry of Health (MoH) and the District authorities in charge of the ART facilities. Confidentiality was upheld throughout the research period.

### Statistical analysis

Data from the data collection form was entered in an access database and later exported to SPSS version 20 and STATA version 12 for analysis. Quantile regression was used to determine the association between CD4 count at 6 and 24 months (dependent variables) and the independent variables. Quantile regression analysis was chosen because the outcome variable (CD4 count) was discrete and not normally distributed. The median was used to assess associations of the dependent variable with the independent variables adjusted for baseline CD4 count at the time of restarting ART. Clustering effect was taken into consideration by adjusting for the different ART facilities were data was collected. Statistical comparisons were made within the groups [24].

## Results

### Characteristics of study participants

The study reviewed 535 records of patients who defaulted and restarted ART at the five ART facilities. Of the 535 patients, 71 (13.3 %) were from Chipokota Mayamba, 89 (16.6 %) from Kabwe General Hospital, 119 (22.2 %) from Kitwe Central Hospital, 31 (5.8 %) from Mwachisompola Demo Zone and 225 (42.1 %) Liteta Hospital. The socio-demographic characteristics of the 535 patients restarted on ART are shown in Tables 1 and 2. More than half of the participants 56.6 % (303) were female while 43.4 % (232) were male. The median age of the patients was 37 years (Interquartile range [IQR] 31 to 43 years) with the minimum age of 17 and the maximum age of 65 years. More than forty percent (43.0 %, *n* = 230) of these patients were falling in the age group 35–44 years. In terms of education, 19.3 % (103) of the patients had no education, 61.1 % (327) had attained some education (ranging from primary to secondary education) while the remaining 16.4 % (88) had attained tertiary education (college or university). In addition, 36.4 % (*n* = 195) of the patients were self-employed, 35.5 % (190) were unemployed and 21.3 % (*n* = 114) were in formal employment. Tables 1 and 2 also shows that 84.3 % of the patients were in WHO stage 3 (*n* = 245; 46.2 %) and WHO stage 4 (*n* = 202; 38.1 %) at the time of restarting ART.

**Table 1:** Socio-demographic and Clinical Characteristics for 535 Patients at ART Restart

		Frequency	Percentage
Sex	Male	232	43.4
	Female	303	56.6
Marital Status	Never Married	77	14.4
	Married	321	60.0
	Divorced	65	12.1
	Widowed	60	11.2
	Missing	12	2.2
Age Group	15–34	148	27.7
	35–44	230	43.0
	45+	154	28.8
	Missing	3	0.6
Level of Education	None	103	19.3
	Highest Grade (1–12)	327	61.1
	College/university	88	16.4
	Missing	17	3.2
Occupation	Unemployed	190	35.5
	Self-employment	195	36.4
	Formal Employment	114	21.3
	Other	14	2.6
	Missing	22	4.1
WHO staging	Stage 4	202	38.1
	Stage 3	245	46.2
	Stage 2	69	13.0
	Stage 1	14	2.6

**Table 2:** Socio-demographic and Clinical Characteristics for 535 Patients at ART Restart

	Median (IQR)	Minimum	maximum
Weight (Kg)	55 (48–61)	20	100
CD4 count	213 (132–306)	1	1556

**Fig. 1:**
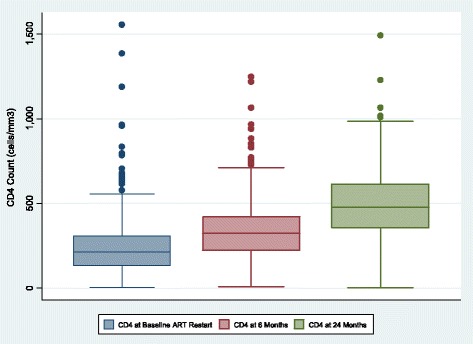
Boxplots of Interquartile range of CD4 count at restarting ART, 6 and 24 months after restaring ART

Figure 1 shows boxplots of interquartile range of CD4 count at restart of ART, 6 and 24 months after restarting ART. The median CD4 count at restart of ART was 213 (IQR, 132 to 306), 323 (IQR, 221 to 420) at 6 months and 476 (IQR, 355 to 612) at 24 months after restarting of ART.

### Quantile regression at 6 months

Quantile regression analysis at 6 months after restarting ART showed that age, occupation, type of treatment, WHO staging, CD4 count, total duration on treatment and duration of LTFU were significantly associated with CD4 count at both bivariate and multivariable analysis (Table 3). Age group and occupation were the only socio-demographic characteristics that predicted CD4 count cell response at 6 months. Patients in the age group ≥45 years had a significantly lower CD4 count at 6 months by 7.9 cells/mm^3^ (95 % CI: −48.1 to −2.7) compared to patients in the age group of 15 to 34 years. In terms of occupation, patients had a significantly higher CD4 count at 6 months if they were in formal employment (Adjusted Coefficient [AC] 29.5, 95 % CI: 22.8 to 81.1) compared to patients who were unemployed; and patients in self-employment showed a significant increase in CD4 cell count by 26.4 cells/mm^3^ (95 % CI: 12.2 to 64.9) compared to patients who were unemployed.

**Table 3:** Quantile logistic regression model on CD4 count of the patients at 6 and 24 months after restarting ART

Independent Variable		Median CD4			
		At 6 Months		At 24 Months	
		UC^a^ (95 % C.I)	AC^b^ (95 % C.I)	UC^a^ (95 % C.I)	AC^b^ (95 % C.I)
Sex	Male	1	1	1	1
	Female	70.3 (48.2;91.7)	19.8 (−10.9;50.4)	37.7 (−25.1;99.2)	19.5 (−65.1;104.2)
Age Group	15–34	1	1	1	1
	35–44	18.5 (2.3;48.8)	20.3 (−1.6;49.7)	16.8 (−56.6;88.5)	−24.2 (−119.4;71.6)
	45+	−10.7 (−55.3;−5.3)	−7.9 (−48.1;−2.7)	18.2 (−63.7;99.8)	−38.1 (−109.4; −0.2)
Occupation	Unemployed	1	1	1	1
	Self-employment	48.9 (18.6;77.4)	26.4 (12.2;64.9)	18.9 (−46.3;82.5)	−5.3 (−112.3;101.6)
	Formal employment	18.1 (−16.3;52.2)	29.5 (22.8;81.1)	24.0 (−51.8;99.5)	4.7 (−130.6;139.9)
	Other	120.2 (38.8;84.9)	104.0 (7.4;200.5)	41.9 (−101.8;183.2)	3.1 (−230.6;236.8)
Education	None	1	1	1	1
	Highest Grade (1–12)	16.0 (−28.6;60.6)	26.4 (−16.3;69.4)	33.1 (−39.9;105.1)	−8.3 (−125.1;108.5)
	College/University	−5.6 (−59.7;49.7)	−0.2 (−64.8;64.4)	15.6 (−77.3; 107.8)	21.3 (149.6;192.2)
Marital status	Unmarried	1	1	1	1
	Married	10.7 (−34.4;54.4)	−9.3 (−52.9;34.4)	−20.7 (−108.9;68.4)	39.7 (−96.5;175.9)
	Divorced	13.6 (−46.1;72.1)	−8.0 (−65.7;49.6)	44.3 (−66.5;154.0)	20.1 (−144.1;184.3)
	Widowed	−14.0 (−75.5;47.5)	−5.1 (−66.1;55.8)	−2.9 (−116.4;112.0)	35.7 (−136.4;207.7)
Type of treatment	1^st^ line	1	1	1	1
	2^nd^ line	−80.2 (−140.4;−19.6)	−65.2 (−127.8;−2.5)	−78.8 (−186.8;30.6)	10.8 (−156.1;177.8)
WHO Staging	Stage 1	1	1	1	1
	Stage 2	−27.4 (−64.3; −10.3)	−18.5 (−59.3;−9.2)	−45.1 (-145.6;−5.7)	−4.7 (−85.7; 14.1)
	Stage 3	−106.2 (−252.5;−68.5)	−116.2 (−230.5;−50.9)	−67.4 (−113.5;−2.3)	−30.0 (−148; 6.1)
	Stage 4	−132.1 (−281.3;−77.9)	−147.8 (−267.5;−31.8)	−97.8 (−183.2;−7.9)	3.4 (−145.6;152.4)
Facility	Chipokota Mayamba Clinic	1	1	1	1
	Kabwe General Hospital	21.7 (−25.0;67.3)	11.1 (−41.8;32.1)	15.7 (5.6;157.5)	−15.4 (−185.6;154.7)
	Kitwe Central Hospital	−34,6 (−82.9;14.7)	−27.3 (−85.7;31.0)	−4.5 (−94.4;88.9)	−57.9 (−225.3;109.4)
	Liteta Hospital	24.5 (−20.3;68.0)	11.2 (−37.4;59.8)	70.3 (−13.8;153.4)	15.6 (−131.2;162.5)
	Mwachisompola Demo Zone	48.3 (−30.3;126.2)	24.6 (−62.9;111.9)	121 (−7.2;249.9)	64.1 (−248.1;376.3)
CD4 Count	CD4 count at restart	0.2 (−0.1;03)		0.4 (0.3;0.5)	
	At 6 months			0.9 (0.7; 1.1)	0.9 (0.5;1.1)
Total Duration on treatment		0.4 (0.1;0.8)	1.2 (0.5;3.7)	0.2 (−1.2;1.6)	0.2 (0.1;0.4)
Duration LTFU		−0.05 (−0.7;0.8)	−1.3 (−1.9;−0.9)	−0.1 (−1.9;1.6)	1.9 (−4.2;8.2)
Duration on treatment before LTFU		0.2 (−1,1;1.5)	1.4 (0.5;3.5)	I.6 (−2.0;4.1)	0.5 (−4.6;5.5)

^a^Unadjusted Coefficient (UC); ^b^Adjusted coefficient (AC), adjusted for sex, age group, occupation, education level, marital status, occupation, type of treatment, duration and CD4 count

The results show that the patients had a significantly lower CD4 count at 6 months if they were receiving second line ART compared to those receiving first line ART; the CD4 was lower by 65.2 cells/mm^3^ (95 % CI: −127.8 to −2.5). The patients also showed a significantly lower CD4 count at 6 months if they were in WHO stage 4 (AC 147.8, 95 % CI: −267.5 to −31.8); in WHO stage 3 (AC 116.2, 95 % CI: −230.5 to −50.9) and in WHO stage 2 (AC18.5, 95 % CI: −59.3 to −9.2) compared to those in WHO stage 1 (Table 3). Furthermore, one-month increase in total duration on treatment significantly increased the CD4 count at 6 months by 0.4 cells/mm^3^ (95 % CI: 0.1 to 0.8).

Duration on treatment before LTFU and duration of LTFU were only significantly associated with CD4 count at 6 months at multivariable analysis. One-month increase in duration LTFU significantly decreased the CD4 count at 6 months by 1.3 cells/mm^3^ (95 % CI: −1.9 to −0.9) while duration before LTFU significantly increased the CD4 count by 1.4 cells/mm^3^ (95 % CI: 0.5 to 3.5). At bivariate analysis, one unit increase in CD4 count at ART restart increased the CD4 count at 6 months by 0.2 cells/mm^3^, with a range of 0.1 cells/mm^3^ to 0.3 cells/mm^3^.

### Quantile regression at 24 months

Using CD4 count at 24 months as the dependent, three independent variables were statistically significant and these were age group, total duration and CD4 count at 6 months. The age group of ≥45 years showed a significantly lower CD4 count by 38.1 cells/mm^3^ (95 % CI: −109.4 to −0.2) compared to the age group of 15–34 years. One unit increase in CD4 count at 6 months increased the CD4 count at 24 months by 0.9 cells/mm^3^, with a range of 0.5 cells/mm^3^ to 1.1 cells/mm^3^. One month increase in total duration on treatment increased the CD4 count at 24 months by 0.2 cells/mm^3^ (95 % CI: 0.1 to 0.4).

At bivariate analysis, CD4 count at restart of ART, WHO staging and facility were significantly associated with CD4 count at 24. One unit increase in CD4 count at restart of ART increased the CD4 count at 24 months by 0.4 cells/mm^3^ (95 % CI: 0.3 to 0.5). The patients consistently showed a significantly lower CD4 count if in WHO stage 4 (UC −97.8, 95 % CI: −183.2 to −7.9), in WHO stage 3 (UC −67.4, 95 % CI: −113.5 to −2.3) and in WHO stage 2 (UC −45.1, 95 % CI: −145.6 to −5.7) compared to those in WHO stage 1. Patients from Kabwe general hospital showed a significant increase in CD4 count at 24 months by 15.7 cells/mm^3^ (95 % CI: 5.6 to 157.5) than the other facilities.

## Discussion

The study demonstrates that different individuals will exhibit different responses in CD4 cell count after restarting ART. Age, occupation, type of treatment, total duration on treatment, duration of LTFU, duration on treatment before LTFU and previous CD4 count were associated with CD4 cell count at 6 months after restarting ART. At 24 months after restarting ART, three (3) variables were significantly associated with CD4 count and these were Age, CD4 count at 6 months and total duration on treatment.

In this study, most of the patients who restarted ART were ≥35 years. The patients in the age group of ≥45 years showed a significant lower CD4 count at 24 months as compared to the age group 15–34 years. Younger age may be associated with better CD4 cell count recovery while older age maybe associated with a weaker immunity system, which makes the immunological recovery slower. The older patients may also be more likely to develop more severe opportunistic infections associated with old age than the younger age group even after they resume ART. This result is consistent with the study done on adults in South Africa, which found that resumption of ART was more likely in patients ≥30 years old than those who are younger [18]. Furthermore, in a cohort study conducted in a hospital setting in Brazil, patients over 40 years of age were reported to present lower increase in CD4 cell count over time compared to the younger age group [25]. This result may have important public health implications in the administration and retention of older patients in ART care.

Patients in formal and self-employment showed a higher CD4 cell count increase as compared to those unemployed and this higher CD4 count was attained in the first year of restarting ART. It is a well-known fact that paid work is good for health and may improve access to health services (including ART care) through employer-sponsored health programmes [26]. The income status of an individual and the family dynamics have been reported to influence the health outcome of the patients on ART. Poor living conditions and failure to meet the indirect cost of ART were factors preventing patients from complying with treatment. As a result, patients with less income have been reported to be 10 times more likely to experience negative life events than patients with high income [26, 27]. This could be the reason why patients in formal and self-employment showed a higher CD4 count increase in this study. In line with this assumption, approaches that aim at improving ART care services must include interventions that deliberately focus on ensuring high levels of adherence and low LTFU. These interventions must be tailored differently and specifically for employed and non-employed patients.

In this study, there was no significant association between education level and CD4 count response at both 6 and 24 months after restarting ART. This could be because the definition for highest grade one to twelve (1–12) in the secondary data used was too broad ranging from primary (grade one) to secondary education (grade 12). Patients who had attained grade one level of education may not be different from those with no education.

In addition to the socio-demographic characteristics associated with CD4 count cell response; CD4 count at restart of ART and WHO stage of the patient were associated with CD4 count cell response at 6 and 24 months after restarting ART. The median CD4 count of the patients restarted on ART was <350 cells/mm^3^ and most of the patients were in WHO stage 4. According to the Zambian National ART Guidelines for 2009 and 2010, patients qualified to be initiated on ART if they presented with CD4 count <350 cells/mm^3^ and were in WHO stage three or four. However, patients who had defaulted ART were restarted regardless of their CD4 cell count [28–30]. This eligibility criteria were recommendations for patients who were initiating ART for the first time but not patients who were restarting ART.

As the patients continued on treatment more and more patients moved to WHO stage 1, as a result, over 83.6 % of the patients were in WHO stage 1 and 2 by the end of 24 months. This was expected in the present study as it has been a trend in previous studies [27, 31]. However, results in this study show that despite being on treatment for 24 months, some patients still remained in WHO stage 4 indicating the negative effect of interrupting ART. This may be because treatment interruption in patients may affect the CD4 cell count response negatively after restarting ART [17]. Treatment interruption is also associated with increased risk of disease progression and death [3, 32]. This could explain why the majority of the patients restarting ART were in WHO stage 4 and not the asymptomatic WHO stage 1.

The increment in the number of patients in WHO stage 1 is an indicator that patients who restart ART are likely to respond well to treatment as suggested by the study which was done in rural Zambia and in settings with inadequate HIV treatment available [19, 33]. Similarly, the current study shows that at both 6 and 24 months after restarting ART, the CD4 count of the patients in WHO stage 2, 3 and 4 increased but was inferior to the increase observed in patients who restarted ART in WHO stage 1. This may be due to the reason that baseline CD4 count is one of the most important determinant of subsequent CD4 cell count trajectories in both patients that are initiated on ART for the first time and those that default and restart ART [34]. These results though obtained from two different groups are comparable. It can, therefore, be suggested that there might be no difference in CD4 count responses between patients who restart and those initiated on ART for the first time. However, there is need for further research in this area.

## Conclusion

The results in this study are crucial to understand the social and clinical characteristic of patients who default and restart ART in Zambia. The socio-demographic characteristics show that patients that restart are more likely to be aged 35 years and above, married, and must have gone to either primary or secondary level of education. CD4 cell count of the patients who restart ART was associated with age, occupation, type of treatment, CD4 at ART restart and CD4 count at 6 months after restarting ART. These significant associations were observed as early as 6 months after restarting ART and seemed to be confounding.

The finding of this study must be considered in light of the limitations of this study. Firstly, data was collected retrospectively hence some variables had missing information which caused the sample size to vary at each stage of the analysis. However, the variables and patient files with significant missing data were excluded during data analysis. All the main predictors for the dependent variable were also adjusted for at each stage of analysis thereby minimizing potential bias in the main results due to confounding. Furthermore, the reason as to why the patients at Kabwe General Hospital recorded a significantly higher increase in CD4 count as compared to other facilities is not known because the data was collected retrospectively. Secondly, adherence to ART after restarting was based on the records from the pharmacy pick-up of the medication. Finally, this study was conducted at only 5 ART facilities in the Copperbelt and Central provinces of Zambia which were purposively sampled. Therefore, the results can only be generalized to similar settings but not for the country.

The outcome of the research suggest that, ART as the essential and vital medicine used to the fight against HIV/AIDS, sustain human lives and prevent HIV/AIDS morbidity and mortality can only give the full benefits when patients stop defaulting and those who restart adhere to treatment after restarting.

## References

[1] Piot P, Coll Seck AM (2001). International response to the HIV/AIDS epidemic: planning for success. Bull World Health Organ.

[2] UNAIDS. Access to Antiretroviral therapy in Africa Status report on progress towards the 2015 targets. 2013. Available from: http://www.unaids.org/sites/default/files/media_asset/20131219_AccessARTAfricaStatusReportProgresstowards2015Targets_en_0.pdf. Accessed March 4, 2014.

[3] Egger M, Spycher BD, Sidle J, Weigel R, Geng EH, Fox MP, MacPhail P, van Cutsem G, Messou E, Wood R (2011). Correcting mortality for loss to follow-up: a nomogram applied to antiretroviral treatment programmes in sub-Saharan Africa. PLoS Med.

[4] McGuire M, Pinoges L, Kanapathipillai R, Munyenyembe T, Huckabee M, Makombe S, Szumilin E, Heinzelmann A, Pujades-Rodriguez M (2012). Treatment initiation, program attrition and patient treatment outcomes associated with scale-up and decentralization of HIV care in rural Malawi. PLoS One.

[5] World Health Organization. UNAIDS Worlds Day Report. 2011. Available from:http://www.unaids.org/sites/default/files/en/media/unaids/contentassets/documents/unaidspublication/2011/JC2216_WorldAIDSday_report_2011_en.pdf. Accessed April 2, 2012.

[6] Panel on Antiretroviral Guidelines for Adults and Adolescents. Guidelines for the use of antiretroviral agents in HIV-1-infected adults and adolescents. Department of Health and Human Services. 2012. Available from: http://www.aidsinfo.nih.gov/ContentFiles/Adul-tandAdolescentGL.pdf. Accessed June 16, 2013.

[7] Fox MP, Rosen S (2010). Patient retention in antiretroviral therapy programs up to three years on treatment in sub-Saharan Africa, 2007-2009: systematic review. Trop Med Int Health.

[8] Musheke M, Bond V, Merten S (2012). Individual and contextual factors influencing patient attrition from antiretroviral therapy care in an urban community of Lusaka, Zambia. J Int AIDS Soc.

[9] Odafe S, Torpey K, Khamofu H, Ogbanufe O, Oladele EA, Kuti O, Adedokun O, Badru T, Okechukwu E, Chabikuli O (2012). The pattern of attrition from an antiretroviral treatment program in Nigeria. PLoS One.

[10] Rosen S, Fox MP, Gill CJ (2007). Patient retention in antiretroviral therapy programs in sub-Saharan Africa: a systematic review. PLoS Med.

[11] Government of the Republic of Zambia, Ministry of Health. Adolescents Antiretroviral Therapy protocols 2010. 2010. Available from: http://www.who.int/hiv/pub/guidelines/zambia_art.pdf. Accessed March 4, 2013.

[12] Cantrell RA, Sinkala M, Megazinni K, Lawson-Marriott S, Washington S, Chi BH, Tambatamba-Chapula B, Levy J, Stringer EM, Mulenga L (2008). A pilot study of food supplementation to improve adherence to antiretroviral therapy among food-insecure adults in Lusaka, Zambia. J Acquir Immune Defic Syndr.

[13] Central Statistics Office, Ministry of Health, Tropical Disease Research Centre, University of Zambia, Macro International Inc. Zambia Demographic and Health Survey 2007. In*.* Calverton, Maryland, USA: CSO and Macro International Inc.; 2009.

[14] Sasaki Y, Kakinoto DC (2012). Adherence to antiretroviral therapy during the early months of treatment in rural Zambia: Influence of demographic charecteristics and social sorroundings of patients. Ann Clin Microbiol Antimicrob.

[15] Brinkhof MW, Pujades-Rodriguez M, Egger M (2009). Mortality of patients lost to follow-up in antiretroviral treatment programmes in resource-limited settings: systematic review and meta-analysis. PLoS One.

[16] Miller CM, Ketlhapile M, Rybasack-Smith H, Rosen S (2010). Why are antiretroviral treatment patients lost to follow-up? A qualitative study from South Africa. Trop Med Int Health.

[17] El-Sadr WM, Lundgren J, Neaton JD, Gordin F, Abrams D, Arduino RC, Babiker A, Burman W, Clumeck N, Cohen CJ (2006). CD4+ count-guided interruption of antiretroviral treatment. N Engl J Med.

[18] Kranzer K, Lewis JJ, Ford N, Zeinecker J, Orrell C, Lawn SD, Bekker LG, Wood R (2010). Treatment interruption in a primary care antiretroviral therapy program in South Africa: cohort analysis of trends and risk factors. J Acquir Immune Defic Syndr.

[19] Kimmel AD, Resch SC, Anglaret X, Daniels N, Goldie SJ, Danel C, Wong AY, Freedberg KA, Weinstein MC (2012). Patient- and population-level health consequences of discontinuing antiretroviral therapy in settings with inadequate HIV treatment availability. Cost Effectiveness and Resource Allocation.

[20] Yu JK, Chen SC, Wang K, Chang C, Makombe SM (2007). True outcomes patients on antiretroviral therapy who are “lost to follow-up” in Malawi. Bulleting of the World Health Organisation.

[21] Birungi J, Mills EJ. Can we increase male involvement in AIDS treatment? The Lancet, 376(9749):1302. 10.1016/S0140-6736(10)61918-6 20951892

[22] Dako-Gyeke P, Snow R, Yawson AE. Who is utilizing anti-retroviral therapy in Ghana: An analysis of ART service utilization. International Journal for Equity in Health. 2012;11(1):1-8. 10.1186/1475-9276-11-62 PMC3475040 23072340

[23] Geng EH, Bwana MB, Muyindike W, Glidden DV, Bangsberg DR, Neilands TB, Bernheimer I, Musinguzi N, Yiannoutsos CT, Martin JN (2013). Failure to initiate antiretroviral therapy, loss to follow-up and mortality among HIV-infected patients during the pre-ART period in Uganda. J Acquir Immune Defic Syndr.

[24] Koenker R, Kelvin F (2001). Quantile Regression. Journal of Economic Perspectives.

[25] Montarroyos UR, Miranda-Filho DB, Cesar CC, Souza WV, Lacerda HR, Albuquerque Mde F, Aguiar MF, Ximenes RA (2014). Factors related to changes in CD4+ T-cell counts over time in patients living with HIV/AIDS: a multilevel analysis. PLoS One.

[26] Nachenge JB, Uthman OA, Peltzer K, Richardson LA, Mills EJ, Amekudzi K, Ouedraogo A. Association between antiretroviral therapy adherence and employment status: systematic review and meta-analysis: Bull World Health Organ. 2015;93(1):29-41. doi:10.2471/BLT.14.138149. Epub 2014 Oct 30. 10.2471/BLT.14.138149 PMC4271680 25558105

[27] Bolton-Moore C, Mubiana-Mbewe M, Cantrell RA, Chintu N, Stringer EM, Chi BH, Sinkala M, Kankasa C, Wilson CM, Wilfert CM (2007). Clinical outcomes and CD4 cell response in children receiving antiretroviral therapy at primary health care facilities in Zambia. JAMA.

[28] Ministry of Health Zambia. Antiretroviral Therapy for Chronic HIV Infection in Adults and Adolescents: New ART Protocol. In*.* app.who.int/medicinedocs/documents/s19278en/s19278en.pdf; 2007: 8-12.

[29] World Health Oranization. Rapid Advice: Antiretroviral Therapy for HIV Infection in Adults and Adolescents. 2009. Available from: www.who.int/hiv/pub/arv/rapid_advice_art.pdf?ua=1 . Accessed June 16, 2013.

[30] World Health Organisation Report (2010). Antiretroviral Therapy for HIV Infection in Adults and Adolescents: Recomendations for Public Health Approach 2010 revision.

[31] Reddi A, Leeper SC, Grobler AC, Geddes R, France KH, Dorse GL, Vlok WJ, Mntambo M, Thomas M, Nixon K. Preliminary outcomes of a paediatric highly active antiretroviral therapy cohort from KwaZulu-Natal, South Africa. BMC Pediatr. 2007;7:13. 10.1186/1471-2431-7-13 PMC1847430 17367540

[32] DART Trial team D (2008). Fixed duration interruptions are inferior to continuous treatment in African adults starting therapy with CD4 cell counts < 200 cells/micro. AIDS.

[33] Carlucci JG, Kamanga A, Sheneberger R, Shepaherd BE, Jenkins CA, Spurrier J, Vermund SH (2008). Predictors of adherence to antiretroviral therapy in rural Zambia. J Acquir Immune Defic Syndr.

[34] Nash D, Katyal M, Brinkhof MW, Keiser O, May M, Hughes R, F. D, Wood R, Sprinz E, Schechter M. Long-term immunological response to antiretroviral therapy in low-income countries: a collaborative analysis of prospective studies. AIDS. 2008;22(17):2291–302. 10.1097/QAD.0b013e3283121ca9 PMC2794130 18981768

